# Providing a rest stop during transportation affects the respiratory bacterial microbiota of beef cattle

**DOI:** 10.3389/fcimb.2025.1622241

**Published:** 2025-09-10

**Authors:** Muhammed Salah Uddin, Emmanuel W. Bumunang, Matthew Waldner, Karen S. Schwartzkopf-Genswein, Daniela M. Meléndez, Yan D. Niu, Trevor W. Alexander

**Affiliations:** ^1^ Lethbridge Research and Development Centre, Agriculture and Agri-Food Canada, Lethbridge, AB, Canada; ^2^ Faculty of Veterinary Medicine, University of Calgary, Calgary, AB, Canada

**Keywords:** bovine respiratory disease, feedlot, transportation, rest stop, microbiota

## Abstract

**Background:**

Bovine respiratory disease (BRD) is a significant health concern in beef cattle production, leading to substantial economic losses. In North America, beef cattle are frequently transported over long distances for placement into feedlots. The respiratory microbiota of cattle, including pathogens, can change after feedlot entry. However, there is limited information on how bacteria are impacted when cattle are offloaded for a rest stop during transportation.

**Results:**

This study investigated the effects of a rest stop during transportation on the nasopharyngeal (NP) microbiota of beef cattle. Two separate trials (N = 80 calves per trial) were conducted with treatment groups assigned to rest stop durations of 12 h (Study 1) and 8 h (Study 2), being compared to control animals without a rest stop. In Study 1, cattle were acclimated to a feedlot prior to transportation while in Study 2, cattle were unacclimated. Following transportation and a rest interval, calves were placed into a feedlot and sampled by NP swab periodically for 28 days. Across treatments and time, the most abundant genera included *Mycoplasma*, *Histophilus*, *Mannheimia*, *Pasteurella, Moraxella*, and *Acinetobacter*. In both studies, microbial diversity and structure were not affected by providing a rest stop. However, NP swabs from more sampling time points had elevated levels of the BRD-associated genera *Mannheimia, Histophilus*, and *Mycoplasma* when the microbiota of calves provided rest were compared to animals given no rest.

**Conclusion:**

Based solely on the increased abundance of BRD-associated bacteria, providing a rest stop during transportation may be a risk factor for BRD. However, it was not possible to associate rest stop-induced changes in microbiota with disease outcome due to a low incidence of BRD. Further evaluation using large-scale studies will help define the impact of a rest stop during transportation, on BRD pathogens and incidence in feedlots.

## 1 Introduction

Livestock transportation can impact animal health, welfare, and production costs (Harris et al., 2017). In North America, beef cattle are often transported to feedlots for finishing, with potential delivery and sale through an auction market prior to feedlot placement. Canadian transport regulations indicate that weaned and unweaned calves cannot be confined on a truck for more than 36 and 12 h, respectively, without being provided food, water and rest for a minimum of 8 h (BCRC, 2023; CFIA, 2019). The average transportation duration for cattle in Canada has been estimated to be 22 h from original destination to a rest stop facility, followed by 16 h of additional transportation to a final destination (Meléndez et al., 2022). Transportation can result in stress and injury to livestock (FAO, 2002), with several factors contributing to stress, including the duration of the journey, feed and water limitation, increased energy expenditure to maintain standing balance, and variable environmental conditions (Schwartzkopf-Genswein and Grandin, 2024; Buckham-Sporer et al., 2023). It has been shown that stress responses associated with long distance transport can be reduced by providing a resting period where animals can lie down and are given access to feed and water (Cooke et al., 2013).

Feedlot morbidity and mortality are most commonly related to bovine respiratory disease (BRD), also called shipping fever (Booker et al., 2008). Various stressors, including transportation, have been associated with BRD (Taylor et al., 2010). This disease results in significant economic losses, due to treatment costs and lost productivity (Timsit et al., 2016). Newly received feedlot cattle are most susceptible to BRD and are often administered metaphylactic antimicrobials when deemed high-risk (recently weaned, unknown immunization history, purchased from an auction market). Bacteria including *Pasteurella multocida*, *Mannheimia haemolytica*, *Histophilus somni*, and *Mycoplasma bovis* are frequently implicated in BRD, often in combination (Confer, 2009). These bacteria are opportunistic pathogens and normally reside in the upper respiratory tract, but can proliferate and cause infection in the lungs of animals that have compromised immunity due to stress or primary viral infection (Hodgson et al., 2005). To date, few studies have investigated the impact of a rest stop, during transportation, on the respiratory microbiota of cattle.

In weaned calves transported > 1,600 km, the abundance of *M. haemolytica* increased (Frank and Smith, 1983). In addition, we have recently shown that the upper respiratory tract microbiota experienced increases in several BRD-associated bacterial genera when provided an 8 h rest after 36 h of transportation (Uddin et al., 2023). Stability of the respiratory microbiota is critical to animal health, providing colonization resistance against pathogens (Bogaert et al., 2004; Cho and Blaser, 2012). Thus, transportation has the potential to impact respiratory bacteria in cattle, and potentially susceptibility to BRD, should pathogen proliferation occur. In this study, we aimed to further evaluate the impact of rest during transportation of beef cattle by comparing changes in respiratory microbiota between cattle provided rest, versus control animals that were not given rest. Two separate studies were conducted to evaluate the impact of rest during transportation.

## 2 Methods

Two studies were completed to evaluate the impact of rest stops during transportation on the respiratory bacterial microbiota of beef cattle. These two studies were part of two larger studies assessing the effect of transport duration and rest stops on the health and welfare of beef calves (Meléndez et al., 2020, 2022). Both Study 1 and Study 2 received approval from the Animal Care Committee of the Lethbridge Research and Development Centre (LeRDC) under ACC numbers 1816 and 2011, respectively. The calves were cared for in accordance with the guidelines established by the Canadian Council of Animal Care (CCAC, 2009). In both studies, cattle were transported using model 379 Peterbilt truck and 2018 Merritt feeder cattle tri-axle trailers bedded with wood shavings. The truck drivers transporting the calves during the study had over 25 years of experience transporting cattle and were Canadian Livestock Transporter (CLT) certified.

### 2.1 Study 1: experimental design and sampling

**Figure 1:**
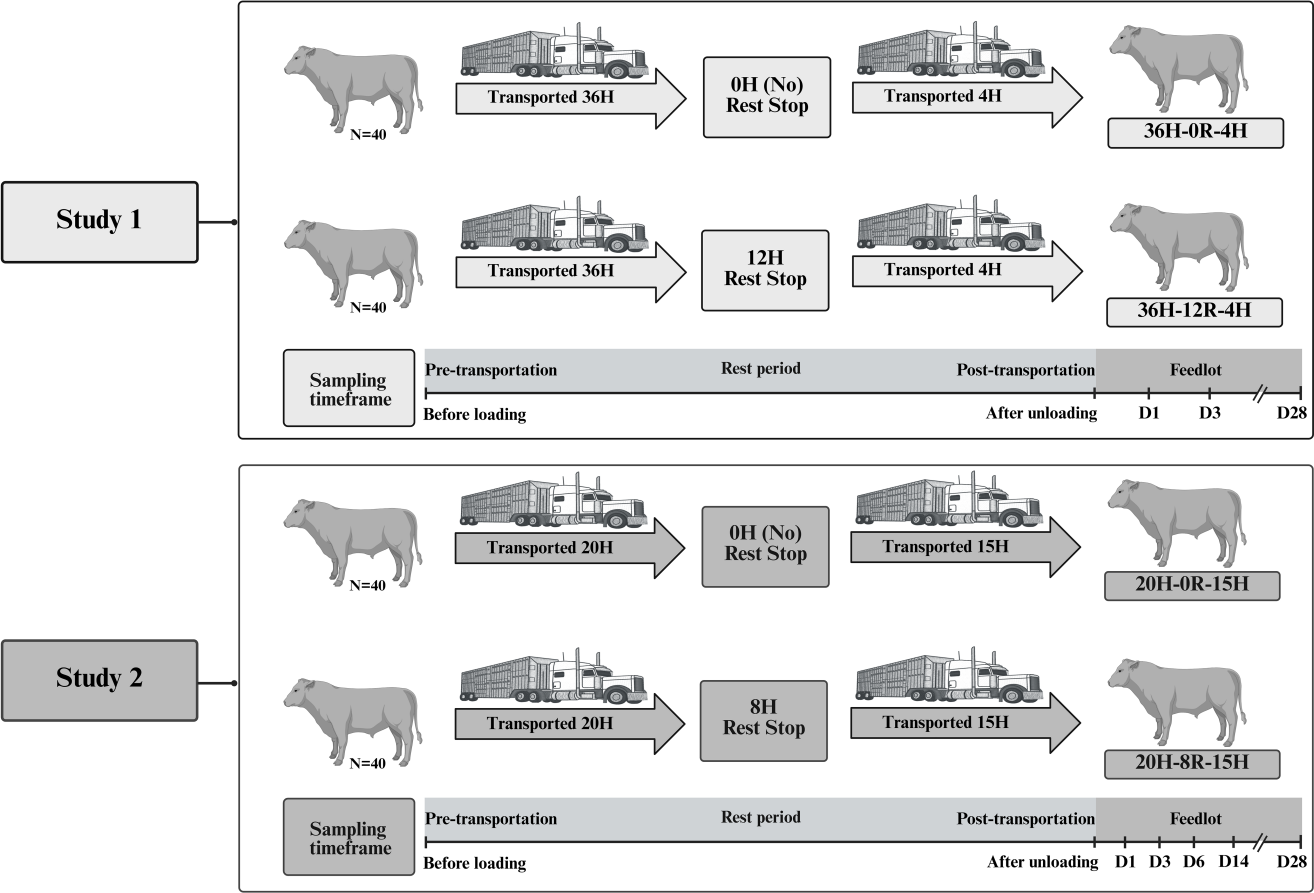
Experimental design. Two separate studies were conducted to evaluate the effect of transport and rest stop duration on the respiratory microbiota of beef cattle. In Study 1, eighty crossbred steer calves were sourced from a ranch in southern Alberta, Canada, and transported to the Lethbridge Research and Development Centre (LeRDC). The calves were conditioned to the feedlot for 26 days and then assigned to no rest (0H) or 12 h of rest (12H) during transportation. All calves were transported 36 h prior to designated rest, then another 4 h afterwards before unloading at the LeRDC feedlot. In Study 2, eighty crossbred steer calves were sourced from two different ranches in southern Alberta, Canada, and transported to the LeRDC. Calves were randomly assigned to no rest (0H) or 8 h of rest (8H) during transportation. All calves were transported for an initial 20 h prior to designated rest, then another 15 h afterwards before unloading at the LeRDC feedlot. In both studies, there were 40 calves in each treatment group, housed in 4 pens, with 10 animals per pen. Three calves per pen were continuously sampled across time. Vertical lines on the sampling timeframe indicate the sampling days for each study; D, day. This figure is created with BioRender.com (Uddin, 2025).

Eighty newly-weaned Black Angus and Black Simmental beef steer calves were transported for approximately 8 h from the ranch of origin to the Lethbridge Research and Development Centre (LeRDC), as described previously (Meléndez et al., 2020). The day after arrival, calves received a 7-way bovine clostridial vaccine (Ultrabac/Somubac, Zoetis Canada Inc., Kirkland, Quebec, Canada); a 5-way bovine viral diarrhea, rhinotracheitis, parainfluenza and bovine respiratory syncytial virus vaccine (Pyramid FP 5 + Presponse SQ, Boehringer Ingelheim, Burlington, Ontario, Canada); an antibiotic (Draxxin, Zoetis Canada Inc., Kirkland, Canada); and an anti-parasitic agent (Ivomec Pour-on for Cattle, Boehringer Ingelheim, Burlington, Ontario, Canada). The calves were vaccinated, ear tagged, and acclimatized for 26 days to eat a grain diet from the feed bunk, and drink from the water trough in the feedlot pens, prior to the start of the trial. This feedlot acclimation was done to reduce the number of stressors experienced by the calves prior to transportation. Calves were then randomly assigned to two treatments and were transported separately for an initial 36 h time period, followed by the assigned rest stop time (0H or 12H), and transported for an additional 4 h in the same trailers for delivery to the LeRDC feedlot. The treatments were (N=40 per treatment): 1) calves with an initial transport of 36 h, followed by 0 h rest and 4 h additional transport, and 2) calves with an initial transport of 36 h, followed by 12 h rest and 4 h additional transport. There were 40 calves in each treatment group, housed in 4 pens (10 animals/pen). A detailed schematic depiction of the experimental design is presented in **Figure 1**.

For each treatment, 12 randomly selected calves (3 calves/pen) were assigned for repetitive sampling. Calves were sampled before loading (BL), after unloading (AU), 1 day AU, 3 days AU, and 28 days AU. Nasopharyngeal (NP) swabs were collected from the right nasal cavity while calves were restrained in a chute. Prior to sampling, the nostril was wiped clean with 70% ethanol. Sterile swabs (27 cm) within a protective outer sheath (MW 124, Medical Wire & Equipment, Corsham, England) were inserted through the nasal passage to the nasopharynx (approximately 70% the head length). The swabs were extended beyond the sheath and rotated in a circular direction for approximately 10 s to collect nasopharyngeal samples as described previously (Holman et al., 2017). Swab tips were then cut and placed into sterile 1.5mL tubes kept on ice. Samples were transported to the lab and stored at -80°C within one hour of collection.

### 2.2 Study 2: experimental design and sampling

Eighty crossbred steer calves (Black or Red Angus × Hereford/Simmental and Black or Red Angus × Charolais) were sourced from two different ranches in southern Alberta, Canada. Upon initial feedlot entry, calves were administered the same vaccines, and antimicrobial as Study 1, and were fed a silage-based diet described previously (Meléndez et al., 2022). However, in Study 2, calves were not acclimated to the feedlot. Calves were randomly assigned to two treatments and were transported separately for an initial 20 h time period, followed by the assigned rest stop time (0H or 8H), and transportation for another 15 h for delivery to the LeRDC feedlot. The treatments were (N=40 per treatment): 1) calves with an initial transport of 20 h, followed by 0 h rest and 15 h additional transport, and 2) calves with an initial transport of 20 h, followed by 8 h rest and 15 h additional transport. A detailed schematic illustrating the experimental design can be found in **Figure 1**.

Each treatment group was comprised of 40 calves, which were housed in 4 pens with 10 animals per pen. Microbial parameters were measured from 12 calves/treatment (3 calves/pen). NP samples were collected before loading (BL), after unloading (AU), and then 1, 3, 6, 14, and 28 days AU, and processed as described previously (Meléndez et al., 2022).

### 2.3 Microbial analysis

Total nucleic acids were extracted from NP swabs as described previously (Holman et al., 2017), including samples collected at all time points from both studies. Briefly, total DNA was extracted from NP swabs using the DNeasy Tissue Kit (Qiagen Inc., Mississauga, ON, Canada), with modifications to optimize bacterial lysis. The cotton tip were removed from the swab and placed in a sterile tube containing 180 µL of enzymatic lysis buffer, which included mutanolysin (300 U/mL) and lysozyme (20 mg/mL). The mixture was vortexed and incubated at 37°C for 1 hour. After enzymatic digestion, 25 µL of proteinase K and 200 µL of Buffer AL (without ethanol) were added to the tube, and the mixture was thoroughly vortexed before being incubated at 56°C for 30 minutes. Approximately 300 mg of sterile 0.1 mm zircon/silica beads were added, and the samples were disrupted using a TissueLyser II (Qiagen) at maximum amplitude for 3 minutes. The samples were then centrifuged at 13,000 × *g* for 5 minutes, and 200 μL of ethanol was added to the supernatants, followed by vortexing. The remaining steps were conducted according to the manufacturer’s protocol for the DNeasy Tissue Kit. In DNA from all swabs, 16S rRNA gene sequence libraries were constructed using a two-step PCR approach. In the first step, the V4 region of the 16S rRNA gene was amplified utilizing primers 515-F (5′-GTGYCAGCMGCCGCGGTAA-3′) and 806-R (5′-GGACTACNVGGGTWTCTAAT-3′). The second PCR step introduced a unique 10-base pair barcode at the 5′ end of each amplicon and incorporated Illumina adapter sequences (Illumina, San Diego, CA, USA). All PCR amplification and sequencing procedures were carried out at Genome Quebec (Montreal, Quebec, Canada). The amplicon was sequenced on a MiSeq instrument (2 x 250 bp with an expected library size of 25,000 reads, Genome Quebec, Montreal, Quebec, Canada) with the MiSeq Reagent Kit v2.

Sequences were analyzed as described previously (Uddin et al., 2023). Briefly, quality verification and summarization of raw reads for V4 amplicons were conducted using FastQC (v0.11.9) and MultiQC (v1.12) (Ewels et al., 2016). Primer removal and trimming of low-quality read regions were performed using Trimmomatic (v0.39) (Bolger et al., 2014) with specific parameters: HEADCROP:18, SLIDINGWINDOW:5:18, LEADING:3, and TRAILING:3. Subsequent statistical analyses were carried out in R (v4.1.0) (R Core Team, 2022). The trimmed paired reads were filtered and merged using DADA2 (v1.22.0) (Callahan et al., 2016). Bimeric sequences were removed with DADA2’s removeBimeraDenovo function, and taxonomic classification was assigned using SILVA 138 database (Quast et al., 2012). Updates to the genus *Mycoplasma* taxonomy were applied manually due to database limitations. Statistical analysis of the data were performed using phyloseq 1.38.0 (McMurdie and Holmes, 2013), vegan 2.5–7 (Oksanen et al., 2019), and DESeq2 1.34.0 (Love et al., 2014). Alpha diversity metrics, including Shannon diversity index and observed richness, were calculated using vegan (v2.5-7) (Oksanen et al., 2019) and visualized with ggplot2 (v3.3.5) (Wickham, 2011). Noise was minimized by filtering the ASV table to retain ASVs present in at least 1% of samples with counts ≥ 2. Two-way ANOVA was used to analyze alpha diversity across treatments and time points. Beta diversity was assessed by normalizing filtered ASV counts using DESeq2’s GMPR size factors, with Bray-Curtis distances calculated and visualized using detrended correspondence analysis (DCA) through phyloseq. Microbial community structure differences were evaluated using PERMANOVA (Adonis function) with 9999 permutations in vegan to identify treatment effects at each time point. Taxonomic abundances at the phylum and genus levels were determined using phyloseq and visualized accordingly. Differentially abundant genera were identified using DESeq2 with a negative binomial model with the following predictors: Treatment + Time + Time: Treatment. Genera with significant differences (P < 0.05, log2(FC) > 2 or <-2) were visualized using heatmaps, detailing both genera-level and ASV-specific changes.

## 3 Results

### 3.1 Morbidity and mortality

In Study 1, during the feeding period, 5 cases of illness were reported. All 5 cases of illness occurred in the 12H rest group, with 4 cases of fever and 1 case of foot rot. In Study 2, two cases of illness were reported throughout the study, both of which were from the 8H rest group. There were no reported cases of mortality in either study.

### 3.2 Study 1: 16S rRNA gene sequencing analysis

The raw ASV table contained 6,623 ASVs with a total of 2,825,036 merged paired reads assigned to 118 samples (data not shown). The median number of sequences per sample was 26,027.5 with a minimum of 0 and maximum of 44,819. After filtering, the ASV table contained 1,244 SVs with a total of 2,409,893 merged paired reads assigned to 116 samples. The median number of sequences per sample was 21,386 with a minimum of 1,178 and maximum of 37,204.

#### 3.2.1 Diversity and structure of the nasopharyngeal microbiota

**Figure 2:**
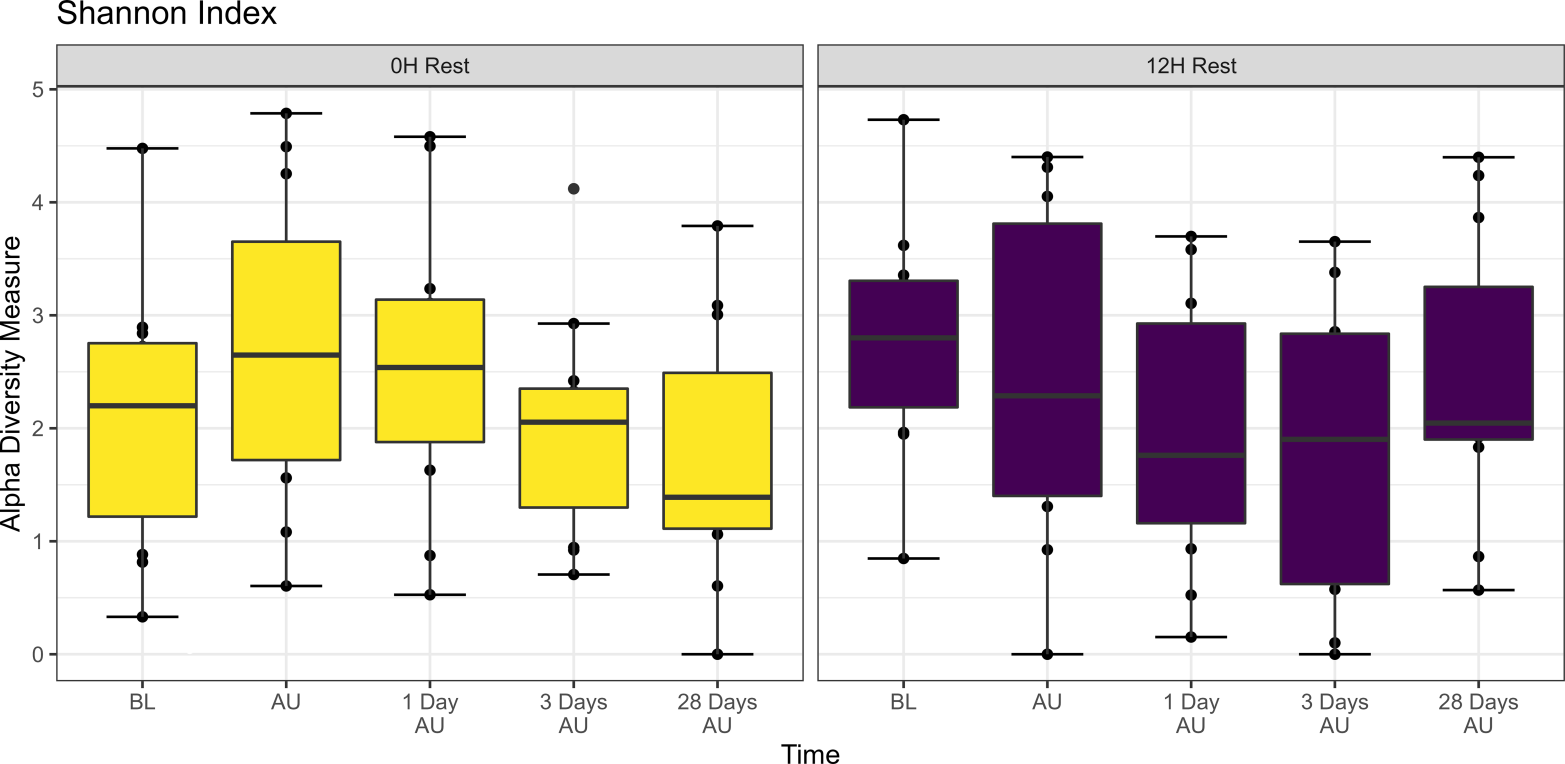
Alpha diversity showing Shannon Index of nasopharyngeal samples from Study 1. Cattle departed from a feedlot and were transported for a total of 36 h and then either provided 0 h (0H Rest) or 12 h (12H Rest) of rest, followed by an additional 4 h of transportation and unloading at the feedlot. Sampling was conducted before loading (BL), after unloading (AU), 1 day AU, 3 days AU, and 28 days AU. The box in the plots indicates the interquartile range (IQR) (middle 50% of the data), the middle line represents the median value, and the whiskers represents 1.5 times the IQR.

The Shannon diversity index was not affected by rest treatment, sampling time, or an interaction between rest and sampling time (P>0.05; **Figure 2**). However, across treatments, there was a trend of increased taxonomic diversity at the AU timepoint when compared to the BL timepoint (P<0.1). PERMANOVA revealed that bacterial structure of the microbiota was weakly affected by rest time during transport (P<0.001, R^2^ = 0.021), and by sampling time (P<0.01, R^2^ = 0.031), but not the interaction of rest and sampling time. DCA plots (**Supplementary Figure 1**) supported PERMANOVA results, with no clear clustering observed by time point.

#### 3.2.2 Composition of the nasopharyngeal microbiota

Across time and treatment groups, a total of 13 different bacterial phyla were identified, among which Proteobacteria (31.4%), Mycoplasmatota (27.8%), Firmicutes (14.8%), Bacteroidota (13.8%), and Actinobacteriota (6.8%) and Euryarchaeota (1.0%) were the most relatively abundant, and together constituted 95.6% of the sequences (**Supplementary Figure 2**). The diversity of genera within each phylum varied, with the relative abundance of a single genus ranging from < 1% to 100% of a phylum. Overall, the 9 most relatively abundant genera across all ASVs, treatments, and time included *Mycoplasma* (27.8%), *Histophilus* (9.8%), *Mannheimia* (3.1%), *Moraxella* (2.4%), *Acinetobacter* (1.1%), *Methanobrevibacter* (1.0%), *Ruminococcaceae* UCG-005 (0.9%), *Psychrobacter* (0.7%), and *Pasteurella* (0.7%) (**Supplementary Figure 3**).

#### 3.2.3 Changes in the microbiota across time and between treatments

**Figure 3:**
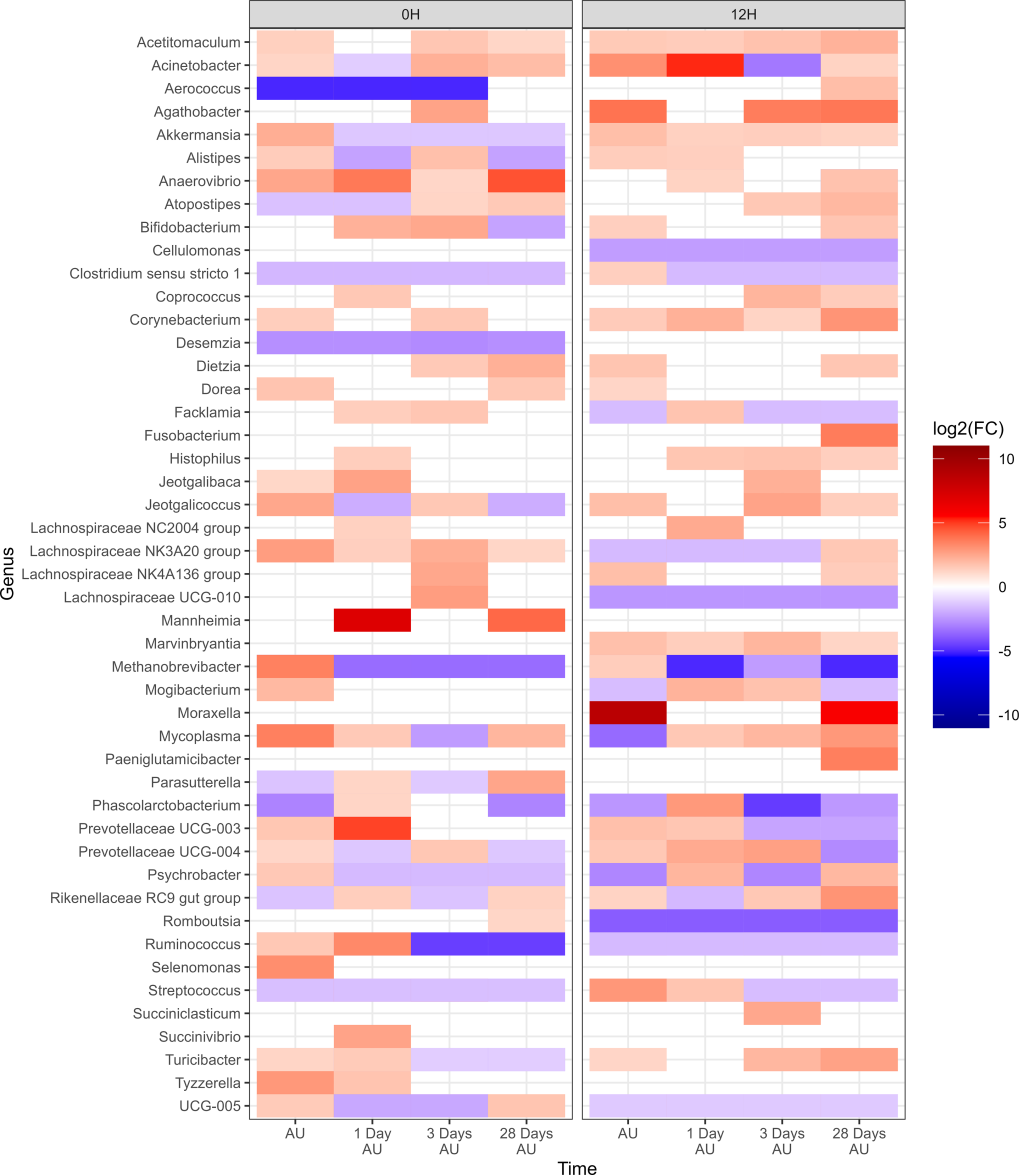
Genera of nasopharyngeal swabs that showed a significant change (P < 0.05, log2(FC) > 2 or log2(FC) < -2) of each sampling time against a baseline time (Before Loading) within the two treatments (0H Rest and 12H Rest) from Study 1. The colors displayed represent the average log2(FC) of amplicon sequence variants (ASVs) with a significant change (P < 0.05) within the respective genus at the indicated time. Cattle departed from a feedlot and were transported for a total of 36 h and then either provided 0 h (0H Rest) or 12 h (12H Rest) of rest, followed by an additional 4 h of transportation and unloading at the feedlot. Sampling was conducted before loading, after unloading (AU), 1 day AU, 3 days AU, and 28 days AU.

Negative-binomial regression was used to identify compositional differences in NP genera across time and treatment groups. In total, 47 genera were identified whose change in relative abundance from the BL sampling time were significant across 0H and 12H rest treatments (P<0.05; **Figure 3**). A total of 41 and 40 genera were identified in the 0H and 12H treatment groups, respectively, that exhibited increases or decreases in relative abundances at subsequent time points compared to the BL time point. Apart from *Pasteurella*, each of the top 9 most relatively abundant genera changed in abundance, with *Acinetobacter*, *Methanobrevibacter*, *Mycoplasma*, *Psychrobacter*, and UCG-005 fluctuating across time for both 0 h and 12 h rest groups. *Histophilus* was increased in relative abundance for at least one time point subsequent to loading, for both 0H and 12H rest group cattle. *Mannheimia* showed the strongest enrichment among genera for the 0H rest group, increasing at 1 and 28 days AU, while *Moraxella* had the strongest increase in abundance AU and 28 days AU in 12H rest cattle.

**Figure 4:**
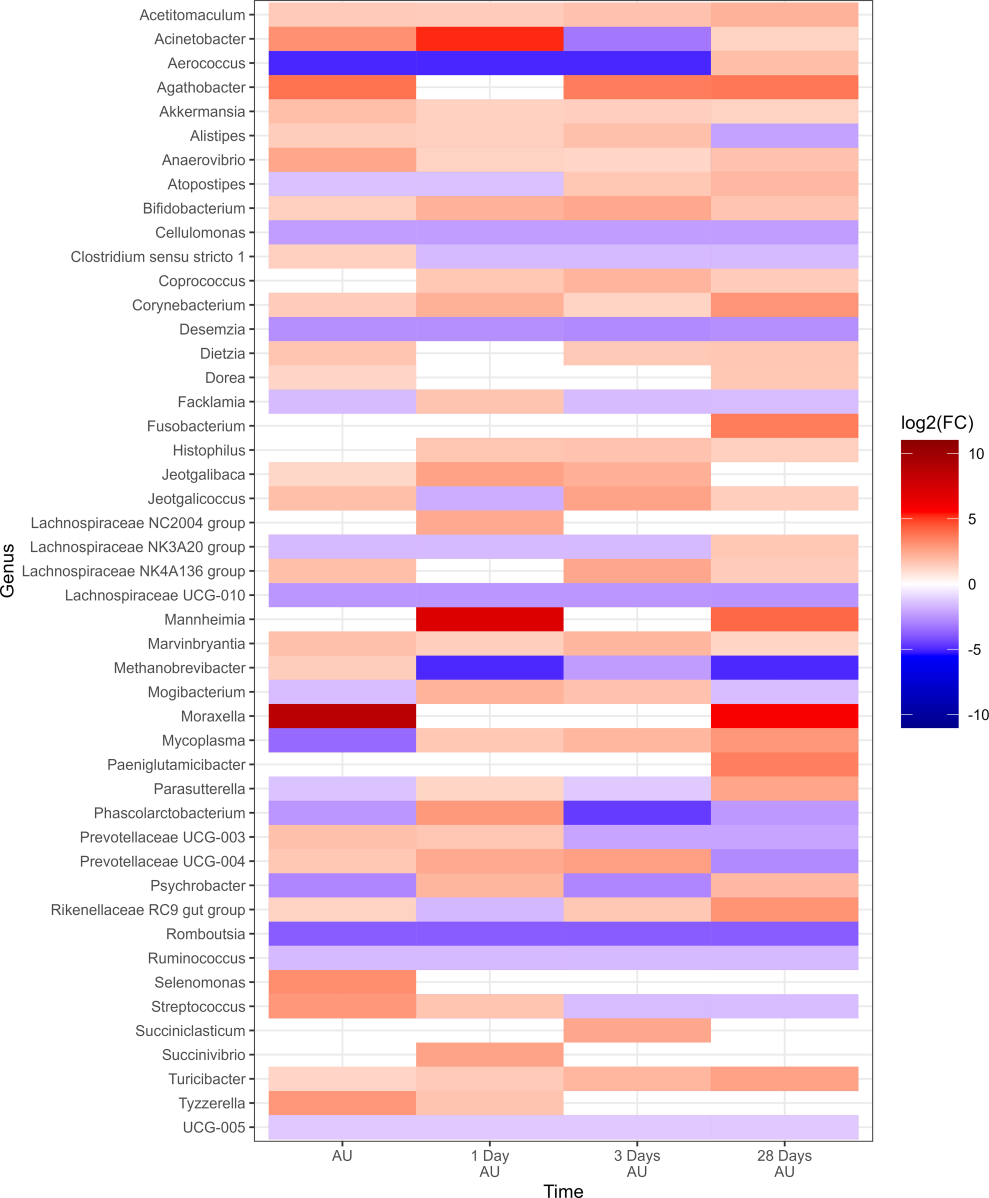
Genera of nasopharyngeal swabs that showed a significant change (P < 0.05, log2(FC) > 2 or log2(FC) < -2) between the treatment “12H Rest” against “0H Rest” with respect to each sampling time against baseline time (Before Loading) from Study 1. The colors displayed represent the average log2(FC) of amplicon sequence variants (ASVs) with a significant change (P < 0.05) within the respective genus at the indicated time. Cattle departed from a feedlot and were transported for a total of 36 h and then either provided 0 h (0H Rest) or 12 h (12H Rest) of rest, followed by an additional 4 h of transportation and unloading at the feedlot. Sampling was conducted before loading, after unloading (AU), 1 day AU, 3 days AU, and 28 days AU.

There were 47 genera within the 12H rest group whose change in relative abundance from the BL sampling time was significantly different from changes that occurred in the 0H rest group (P<0.05; **Figure 4**). Each of the 9 most abundant genera, except for *Pasteurella*, were among these taxa that differed from the 0H rest group. Several genera were shown to consistently remain elevated at every time point (*Acetitomaculum*, *Akkermansia*, *Anaerovibrio*, *Bifidobacterium*, *Corynebacterium*, *Marvinbryantia*, and *Turicibacter*), while others remained consistently lower (*Cellulomonas*, *Lachnospiraceae* UCG-010, *Romboutsia*, *Ruminococcus*, and UCG-005) in relative abundance. Of genera commonly associated with BRD, *Histophilus* was elevated at 1, 3, and 28 days AU, *Mannheimia* was strongly increased at 1 and 28 days AU, *Moraxella* was strongly increased at AU and 28 days AU, and *Mycoplasma* was reduced at AU, then elevated at 1, 3, and 28 days AU. In **Table 1**, BRD-associated genera that differed at each time point AU are listed. The BRD-associated bacteria were elevated in 12H rested cattle at more time points than the 0H rested group.

**Table 1:** Number of time points when BRD-associated genera differed for cattle given 12 h (Study 1) or 8 h (Study 2) of rest during transportation versus cattle given 0 h of rest.

*Genera*	*Study 1^a^*		*Study 2^b^*	
	Increased	Decreased	Increased	Decreased
*Histophilus*	3	–	4	2
*Mannheimia*	2	-	4	2
*Moraxella*	2	–	5	1
*Mycoplasma*	3	1	5	1
*Pasteurella*	–	–	2	2

a Data summarized from comparisons made in **Figure 4**. ^b^Data summarized from comparisons made in **Figure 7**.

### 3.3 Study 2: 16S rRNA gene sequencing analysis

The raw ASV table contained 13,773 ASVs with a total of 4,906,901 reads assigned to 168 samples (data not shown). The median number of sequences per sample was 24,743.5 with a minimum of 4,123 and maximum of 82,673. After filtering, the ASV table contained 324 SVs with a total of 4,016,702 reads. The median number of sequences per sample was 19,528.5 with a minimum of 2,124 and maximum of 79,463.

#### 3.3.1 Diversity and structure of the nasopharyngeal microbiota

**Figure 5:**
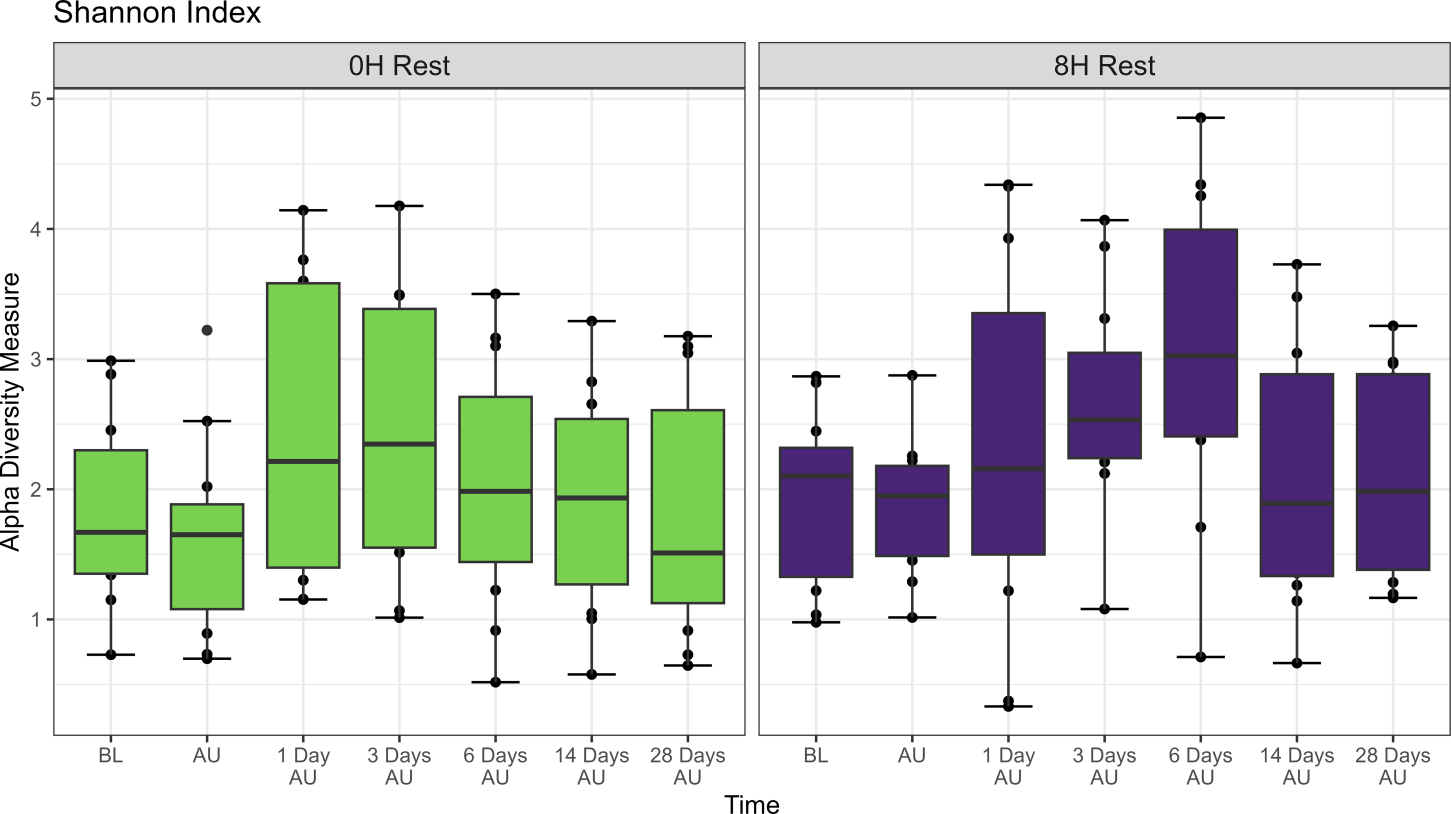
Alpha diversity showing Shannon Index of nasopharyngeal samples from Study 2. Calves from the two treatment groups were loaded onto trailers at the same time, and transported 20 h, and then either provided 0 h (0H Rest) or 8 h (8H Rest) of rest, followed by an additional 15 h of transportation and unloading at the feedlot. Samples were collected before loading (BL), after unloading (AU), and then 1, 3, 6, 14, and 28 days AU. The box in the plots indicates the interquartile range (IQR) (middle 50% of the data), the middle line represents the median value, and the whiskers represents 1.5 times the IQR.

The Shannon diversity index was only affected by sampling time (P<0.05; **Figure 5**), with both rest treatment and the interaction of rest and sampling time having no effect (P>0.05; **Figure 5**). Across treatments, diversity increased at 1, 3, and 6 days AU (P<0.05). PERMANOVA revealed that bacterial structure of the microbiota was affected by sampling time (P<0.001, R^2^ = 0.043), but not by rest time or the interaction between rest treatment and sampling time. A PERMADISP conducted showed that variance in community composition varied significantly within at least one group in the sampling times (P=0.048), but not between the two rest times (P>0.05). DCA plots (**Supplementary Figure 4**) highlighted clustering according to sampling time for both rest treatments.

#### 3.3.2 Composition of the nasopharyngeal microbiota

Across time and treatment groups, a total of 11 different bacterial phyla were identified, among which Proteobacteria (35.3%), Mycoplasmatota (30.7%), Actinobacteriota (21.6%), Bacteroidota (4.2%), Fusobacteriota (3.2%), and Firmicutes (2.7%) were the most relatively abundant, and together constituted 97.7% of the sequences (**Supplementary Figure 5**). The diversity of genera within each phylum varied with the relative abundance of a single genus ranging from < 1% to 100% of a phylum. Overall, the 9 most relatively abundant genera across all ASVs, treatments, and time included *Mycoplasma* (30.7%), *Moraxella* (10.2%), *Pasteurella* (9.7%), *Fusobacterium* (2.7%), *Acinetobacter* (1.4%), *Mannheimia* (1.0%), *Histophilus* (0.7%), *Corynebacterium* (0.6%), and *Caviibacter* (0.6%) (**Supplementary Figure 6**).

#### 3.3.3 Changes in the microbiota across time and between treatments

**Figure 6:**
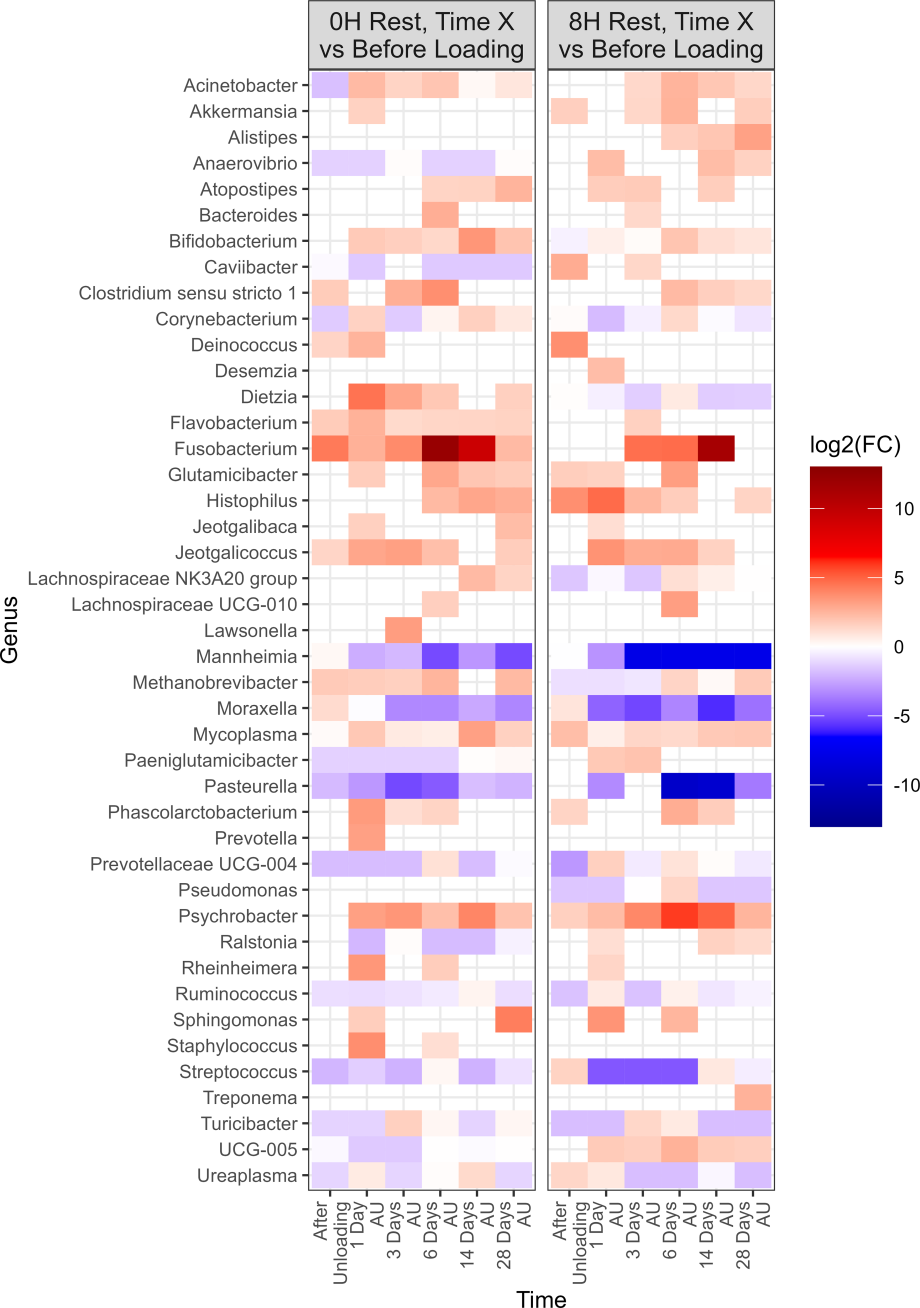
Genera that showed a significant change (P < 0.05, log2(FC) > 2 or log2(FC) < -2) of each sampling time against a baseline time (Before Loading) within the treatments from Study 2. Calves from the two treatment groups were loaded onto trailers at the same time, and transported an initial duration of 20 h. Following the assigned rest stop duration (0 h or 8 h), calves were transported an additional 15 h, and then unloaded at the LeRDC feedlot. Nasopharyngeal samples were collected before loading (BL), after unloading (AU), and then 1, 3, 6, 14, and 28 days AU.

Negative-binomial regression indicated a total of 43 genera exhibited significant changes in relative abundance from the baseline sampling time across both 0H and 8H rest treatments (P<0.05; **Figure 6**). A total of 39 genera were identified in the 0H treatment group, showing variations in their relative abundances at subsequent time points in comparison to the BL time point. Similarly, in the 8H treatment group, 40 genera demonstrated increases or decreases in abundance at later time points when compared to the BL time point. All of the top 9 most relatively abundant genera were among taxa that differed from the BL samples. Several taxa showed similar trends across all treatments. Notably, *Fusobacterium, Glutamicibacter, Histophilus, Jeotgalicoccus, Mycoplasma, and Psychrobacter* showed a consistent increase in relative abundance at various time points subsequent to loading, for both 0H and 8H rested cattle. In contrast, *Mannheimia, Moraxella, and Pasteurella* showed a decrease in relative abundance at the majority of AU time points, across both treatments. UCG-005 was reduced at all time points for 0H calves at the feedlot, while it was increased at most feedlot time points in 8H rested calves.

**Figure 7:**
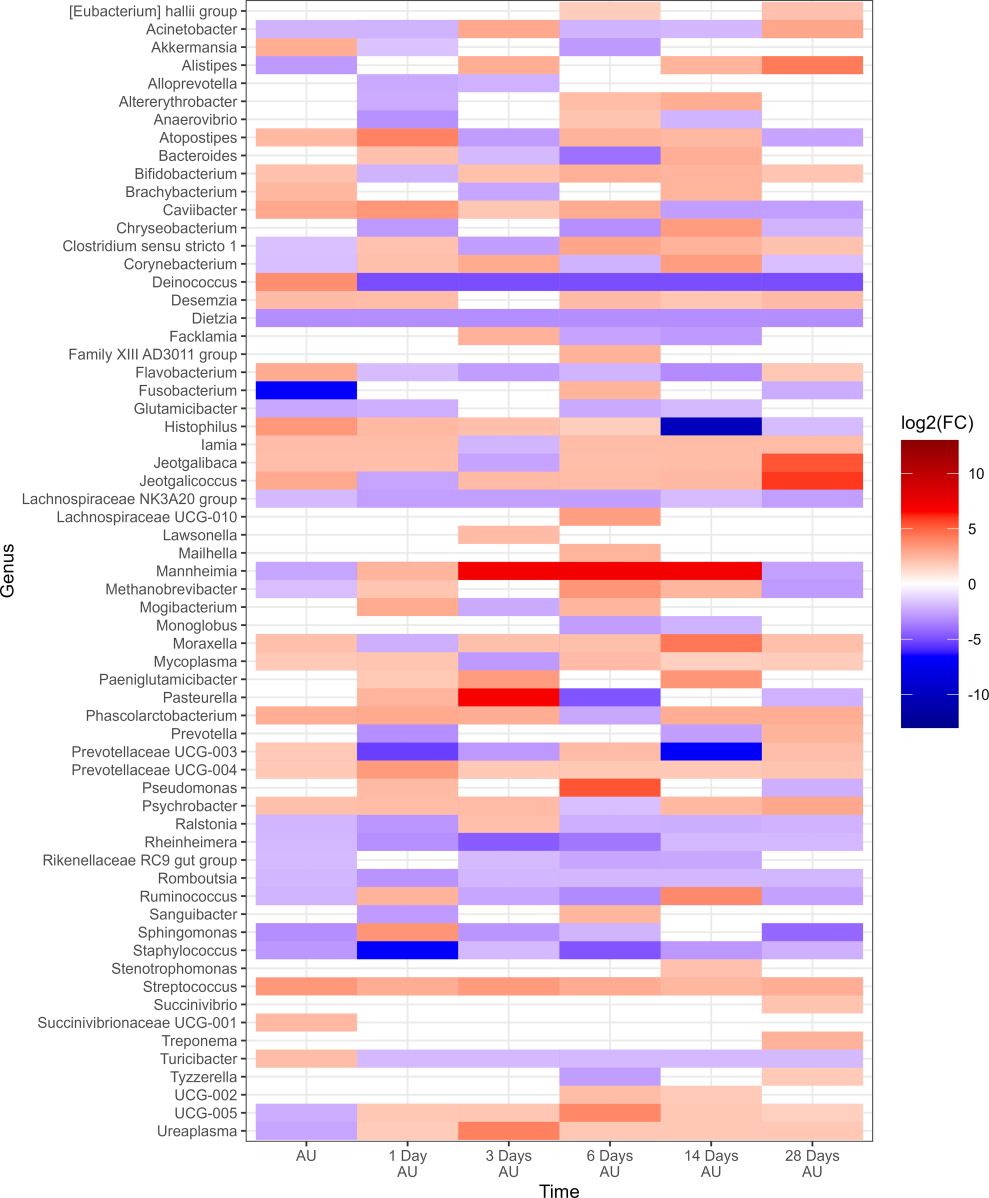
Genera of nasopharyngeal swabs that showed a significant change (P < 0.05, log2(FC) > 2 or log2(FC) < -2) between “8H Rest” vs “0H Rest” with respect to each sampling time vs baseline sampling time (Before Loading) from Study 2. The colors displayed represent the average log2(FC) of amplicon sequence variants (ASVs) with a significant change (P < 0.05) within the respective genus at the indicated time. Calves were loaded onto trailers at the same time, and transported 20 h. Following the assigned rest stop duration (8 h versus 0 h), calves were transported an additional 15 h, and then unloaded at the feedlot. Samples were collected before loading, after unloading (AU), and then 1, 3, 6, 14, and 28 days AU.

To evaluate the impact of rest stop on microbiota of calves, negative-binomial regression comparisons were analyzed for 8H rest against 0H rest calves (**Figure 7**). Sixty-three genera were identified in the 8H treatment group with relative abundances that changed in comparison to the BL time point, to a greater extent than 0H rested calves (P<0.05). *Dietzia*, *Lachnospiraceae* NK3A20 group, *Rheinheimera*, *Romboutsia*, and *Staphylococcus* showed consistent reductions across time in 8H calves. In contrast, *Streptococcus* was consistently enriched in 8H calves at every time point, compared to 0H rest calves. *Mannheimia, Histophilus, Mycoplasma and Moraxella* were all enriched at four or more out of six time points in 8H rested calves, compared to 0H rested calves. Genera associated with BRD, that differed at each time, are shown in **Table 1**. Similar to Study 1, BRD-associated bacteria were elevated at more time points in 8H rest calves, compared to the 0H rest group.

## 4 Discussion

Longer transportation distances or sale through an auction market can increase the relative abundance of BRD-related pathogens (Chai et al., 2022; Uddin et al., 2023) and the incidence of BRD (Cernicchiaro et al., 2012; Padalino et al., 2021) in beef calves. Thus, the effect of transportation on the bovine NP microbiota, particularly following a rest stop, is important for understanding the increased vulnerability of beef cattle to BRD after transport to feedlots. Two studies were therefore conducted, to evaluate how rest during transportation impacts the upper respiratory tract bacteria of feedlot calves. In Study 1, calves were first acclimated to a feedlot setting, to limit the effect of feedlot placement stressors on calf microbiota (Meléndez et al., 2020), while in Study 2, no acclimation was provided (Meléndez et al., 2022). A limitation of this research was that different transportation distances and rest intervals were investigated in Studies 1 and 2. In addition, the sample size in each study was not large enough to evaluate whether BRD incidence was impacted by rest stop interval or changes in microbiota, as too few animals were diagnosed with BRD. Lastly, within treatment, cattle were transported together on the same trailer due to logistical challenges and costs that would be required to rent multiple transporters. However, despite these limitations, there were commonalities between Studies 1 and 2 of the current study, and a previous report publication (Uddin et al., 2023), on how the respiratory microbiota were impacted by transportation rest.

### 4.1 Diversity of the nasopharyngeal microbiota

The characteristics of a bacterial community within a specific niche include the number of species present, their numerical composition, and bacterial diversity (Kim et al., 2017). The NP microbial diversity did not change in Study 1, while in Study 2, it increased across feedlot sampling, mainly due to increased presence of less abundant taxa. Similar to Study 2, previous publications have demonstrated an increase in the NP bacterial and archaeal diversity after cattle are placed in a feedlot (Holman et al., 2017; Amat et al., 2019). This was likely due to the feedlot acting as a source of bacteria that colonized the respiratory tract of calves in Study 2 via aspiration. In addition, all calves received tulathromycin upon arrival at the feedlot as part of a standard management practice aimed at mitigating BRD. It has been reported that while tulathromycin can reduce the total bacterial load in the nasopharynx of cattle, its administration increases NP bacterial diversity (Amat et al., 2023). Thus exposure to the feedlot and antimicrobial administration likely selected for an increase in microbial diversity in Study 2. In contrast, these selection pressures were already experienced 26 days prior by calves in Study 1, due to feedlot acclimating, limiting their effect after transportation and unloading in Study 1 animals.

### 4.2 Composition of the nasopharyngeal microbiota over time

Across both studies, the predominant bacterial phyla were consistent with previous publications investigating NP bacteria of feedlot calves (Timsit et al., 2016; Holman et al., 2017; Stroebel et al., 2018) and included Proteobacteria, Mycoplasmatota, Actinobacteriota, Bacteroidota, and Firmicutes. However, variations have been noted previously and were present in Studies 1 and 2. For instance, Firmicutes accounted for 14.8% of relative abundance in Study 1 but only 2.7% in Study 2. Stroebel et al. (2018) reported that Firmicutes made up only 3% of the nasopharyngeal microbiota in newly weaned feedlot heifers derived from auction or cow-calf ranches, while Holman et al. (2017) found a relative abundance exceeding 20% in calves transported to a feedlot. Similarly, while Proteobacteria accounted for 31.4% and 35.3% of the microbiota in Studies 1 and 2, respectively, this phylum has ranged from 16 - 43% relative abundance in NP microbiota form other studies (McMullen et al., 2018; Uddin et al., 2023). These discrepancies show that while certain bacterial phyla consistently dominate the nasopharyngeal microbiota of calves, their relative abundances can vary due to factors such as the feedlot environment, sampling time, diet, and host-related differences (Alexander et al., 2020) including stress.

In both Studies 1 and 2, numerous genera changed in relative abundance after unloading, compared to the baseline before loading, with some genera sustaining increases or decreases across time. Thus, the microbiota of the bovine NP is dynamic and experiences both transient and stable shifts in populations. Notably, several genera associated with the gastrointestinal tract (*e.g. Bififobacterium, Methanobrevibacter, Ruminococcus, and UCG-005)* were present in NP samples, which has been observed previously (Holman et al., 2019). Except for *Fusobacterium*, which typically lines the wall of the rumen (Tadepalli et al., 2009) and sustained increased abundance after unloading in Study 2 calves, most genera associated with the rumen were variable in abundance. This likely highlights their periodic introduction to the upper respiratory tract from the rumen during rumination.

*Mycoplasma, Histophilus*, and *Acinetobacter* were three of the most relatively abundant taxa and mainly increased after unloading in both Studies 1 and 2. These genera are often reported to be part of the dominant NP genera and increase after feedlot placement (Holman et al., 2017; Zeineldin et al., 2017; Holman et al., 2018; Stroebel et al., 2018). In both studies, bacteria associated with BRD (*Mannheimia, Pasteurella, Histophilus*, and *Mycoplasma*; Booker et al., 2008; Confer, 2009), were part of the 9 most abundant genera, as was *Moraxella*, which has been shown to increase in cattle that develop bronchopneumonia (McMullen et al., 2020). In Study 1, *Mannheima* and *Moraxella* were variable between rest treatments but were observed to increase, while *Pasteurella* did not change after unloading. In contrast, from one day after unloading, and onwards, each of these genera were persistently reduced in both rest treatment groups in Study 2. The reduction was likely due to the administration of tulathromycin shortly before collection of NP swabs in Study 2 animals. Tulathromcyin administration has been observed to reduce prevalence or abundance of *Mannheimia*, *Pasteurella*, and *Moraxella* in the nasopharynx of cattle (Holman et al., 2019; Uddin et al., 2023; Amat et al., 2023).

### 4.3 The effect of rest stop on BRD-associated bacteria

BRD is polymicrobial in nature but genera *Histophilus, Mannheimia*, *Mycoplasma*, and *Pasteurella* are most often associated with infection (Booker et al., 2008; Confer, 2009; Timsit et al., 2017). *Mannheimia* and *Pasteurella* can be the primary cause of fatal secondary infections after feedlot arrival (Malmuthuge et al., 2021) but *Histophilus* is increasingly being implicated and presents later in feedlot placement (Timsit et al., 2017). While not typically associated with bronchopneumonia, *Moraxella* has been observed as a potential risk factor for BRD in received cattle (McMullen et al., 2020) thus was included in our summary of BRD-associated bacteria.

Overall, calves provided rest during transportation were more likely to have increases in abundances of these genera, at more time points after unloading, compared to those provided no rest. This was evident for both feedlot-acclimated (Study 1) and unacclimated (Study 2) calves. Interestingly, in Study 2, there was a sustained increase in *Streptococcus* abundance in calves provided rest which has been observed previously (Uddin et al., 2023). Although the impact on respiratory health is unknown, this genus encodes sialidases which degrade mucus glycans (Xu et al., 2008, 2011), and therefore could potentially alter protection conferred by mucus in the respiratory tract. Despite colonization by these genera in animals across both rest stop treatments, and healthy and BRD-afflicted cattle in other studies (Timsit et al, 2018), their abundance is important to BRD progression. Enrichment of *Mannheimia* and *Pasteurella* in the nasopharynx and lung of BRD morbidities has been observed (Timsit et al., 2018; Centeno-Martinez et al., 2022), and *M. haemolytica* prevalence has been associated with increased risk of BRD in cattle sampled at feedlot entry (Noyes et al., 2015). The same observations reported here, were also found in our previous study showing rest stop to be associated with increases in *Histophilus, Mannheimia*, *Mycoplasma, Pasteurella*, and *Streptococcus* (Uddin et al., 2023). However, unlike Uddin et al. (2023) we did not observe a reduction in commensal *Lactobacillus* due to a rest stop. While *Lactobacillus* can be negatively associated with *Pasteurellaceae* (Amat et al., 2019), their abundance can vary in cattle. Overall, based solely on increases in abundance of BRD-associated genera in the nasopharynx of calves, providing rest during transportation may be a risk factor for BRD.

Stress during transportation is typically evaluated using physiological and behavioral metrics, with circulating cortisol levels serving as a primary indicator of hypothalamic-pituitary-adrenal axis activation (Buckham-Sporer et al., 2023). Elevated cortisol concentrations have been consistently observed in cattle transport studies (Gupta et al., 2007; Odore et al., 2004; Buckham-Sporer et al., 2007), peaking at the start of the journey (Agnes et al., 1990; Fell and Shutt, 1986; Sartorelli et al., 1992), suggesting that handling during loading is the most stressful phase (Agnes et al., 1990; Grigor et al., 2001; María et al., 2004; Pettiford et al., 2008; Buckham-Sporer et al., 2023). This may explain why cattle provided rest had greater increases in BRD-associated genera, with the additional loading and unloading events adding to host stress. Although it has not been defined how stress affects BRD pathogen colonization, we have previously shown blood stress parameters, such as cortisol and haptoglobin, to be associated with bovine NP respiratory bacteria (Uddin et al., 2023) implying a host-bacterial relationship. In addition, while not evaluated in this study, it is possible that rest stations may increase chance of pathogen exposure, as these facilities are routinely utilized by multiple cattle groups over time. Overall, off-loading and reloading for a rest may elevate stress in calves, potentially leading to increased pathogen abundance. While noteworthy that only animals in rested groups were treated for illness, there were too few animals in our studies to support the association between BRD incidence and rest during transportation, and therefore larger scale studies would help define any relationship.

## 5 Conclusion

This research further defined the effect of providing a rest stop during transportation, on the NP microbiota of cattle. Two separate studies were conducted and in both studies, the microbiota of calves were dynamic after unloading, with bacteria being observed as transient or establishing colonization. The microbiota of acclimated calves was more resistant to changes than unacclimated calves. However, in both study groups, the abundances of *Histophilus*, *Mannheimia*, *Moraxella*, and *Mycoplasma* were found to be increased at most time points after transportation, when calves that were provided rest were compared to those given no rest. Based on the increases in BRD-associated genera, these studies suggest that off-loading cattle for a rest during transportation may be a risk factor for BRD in feedlots. Additional studies are needed to further define the relationship between transportation rest, respiratory microbiota, and BRD incidence.

## Data Availability

The datasets presented in this study can be found in online repositories. The names of the repository/repositories and accession number(s) can be found below: https://www.ncbi.nlm.nih.gov/, PRJNA1252120.

## Supplementary material

Supplementary Figure 1 Detrended correspondence analysis (DCA) of the Bray-Curtis metric for each treatment in Study 1. Cattle departed from a feedlot and were transported for a total of 36 h and then either provided 0 h (0H Rest) or 12 h (12H Rest) of rest, followed by an additional 4 h of transportation and unloading at the feedlot. Sampling was conducted before loading (BL), after unloading (AU), 1 day AU, 3 days AU, and 28 days AU.

Supplementary Figure 2 Relative abundance of the six most dominant phyla in the nasopharyngeal samples from Study 1. Cattle departed from a feedlot and were transported for a total of 36 h and then either provided 0 h (0H Rest) or 12 h (12H Rest) of rest, followed by an additional 4 h of transportation and unloading at the feedlot. Sampling was conducted before loading (BL), after unloading (AU), 1 day AU, 3 days AU, and 28 days AU. 16S sequences that were assigned to a phylum lacking enough abundance to be visualized were assigned within the ‘Other’ bar. The ‘Unclassified’ bar represents the relative abundance of 16S sequences that could not be assigned a phylum level.

Supplementary Figure 3 Relative abundance of the nine most abundant genera identified in nasopharyngeal swabs from Study 1. Cattle departed from a feedlot and were transported for a total of 36 h and then either provided 0 h (0H Rest) or 12 h (12H Rest) of rest, followed by an additional 4 h of transportation and unloading at the feedlot. Sampling was conducted before loading (BL), after unloading (AU), 1 day AU, 3 days AU, and 28 days AU. The box in the plots indicates the interquartile range (IQR) (middle 50% of the data), the middle line represents the median value, and the whiskers represents 1.5 times the IQR.

Supplementary Figure 4 Detrended correspondence analysis (DCA) of the Bray-Curtis metric for each treatment in Study 2. Calves from the two treatment groups were loaded onto trailers at the same time, and transported 20 h. Following the assigned rest stop duration (0 h or 8 h), calves were transported an additional 15 h, and then unloaded at the LeRDC feedlot. Nasopharyngeal swabs were collected before loading (BL), after unloading (AU), and then 1, 3, 6, 14, and 28 days AU.

Supplementary Figure 5 Relative abundance of the six most dominant phyla in the nasopharyngeal samples from Study 2. Calves from the two treatment groups were loaded onto trailers at the same time, and transported 20 h. Following the assigned rest stop duration (0 h or 8 h), calves were transported an additional 15 h, and then unloaded at the LeRDC feedlot. Nasopharyngeal swabs were collected before loading (BL), after unloading (AU), and then 1, 3, 6, 14, and 28 days AU. 16S sequences that were assigned to a phylum lacking enough abundance to be visualized were assigned within the ‘Other’ bar. The ‘Unclassified’ bar represents the relative abundance of 16S sequences that were unable to be assigned a phylum level.

Supplementary Figure 6 Relative abundance of the nine most abundant genera identified in nasopharyngeal swabs from Study 2. Calves from the two treatment groups were loaded onto trailers at the same time, and transported an initial duration of 20 h. Following the assigned rest stop duration (0 h or 8 h), calves were transported an additional 15 h, and then unloaded at the LeRDC feedlot. Samples were collected before loading (BL), after unloading (AU), and then 1, 3, 6, 14, and 28 days AU. The box in the plots indicates the interquartile range (IQR) (middle 50% of the data), the middle line represents the median value, and the whiskers represents 1.5 times the IQR.
