# Supplementary material for: Providing a rest stop during transportation affects the respiratory bacterial microbiota of beef cattle
Source: Front Cell Infect Microbiol. 2025 Sep 10;15:1622241. doi: 10.3389/fcimb.2025.1622241 (PMC12457343; doi:10.3389/fcimb.2025.1622241)
Supplement: Supplementary Figure 1 — Detrended correspondence analysis (DCA) of the Bray-Curtis metric for each treatment in Study 1. Cattle departed from a feedlot and were transported for a total of 36 h and then either provided 0 h (0H Rest) or 12 h (12H Rest) of rest, followed by an additional 4 h of transportation and unloading at the feedlot. Sampling was conducted before loading (BL), after unloading (AU), 1 day AU, 3 days AU, and 28 days AU. [file DataSheet1.pdf]

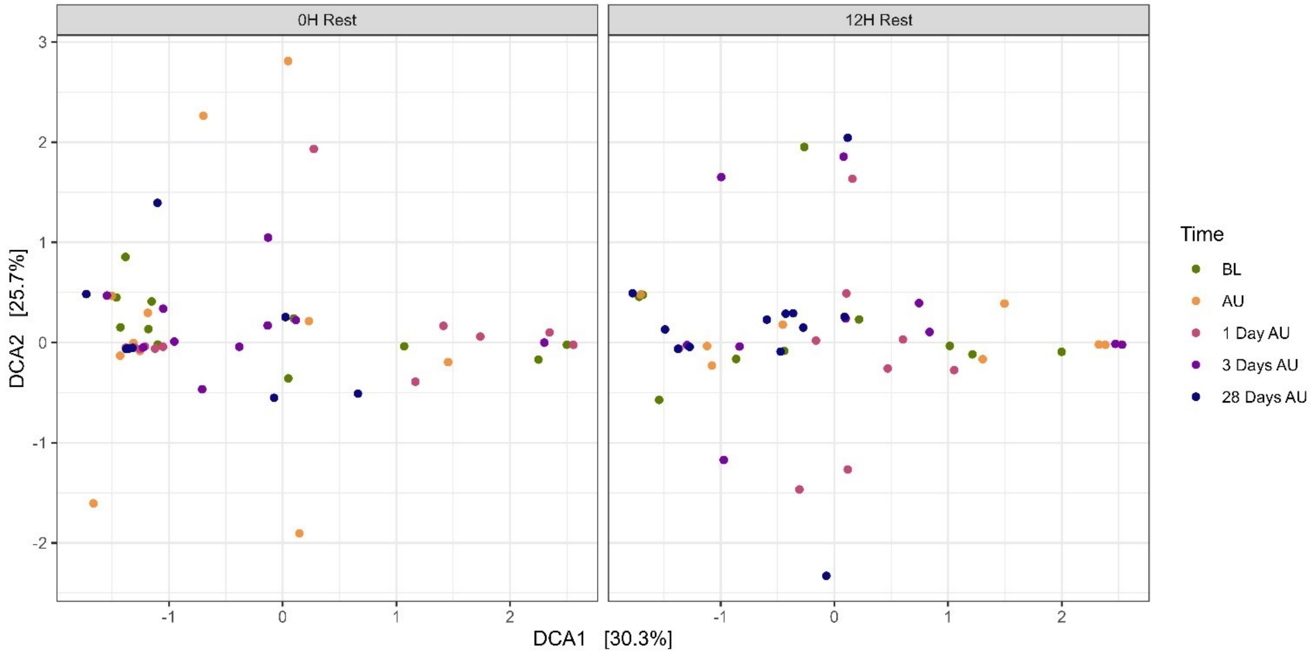

Supplemental Figure 1: Detrended correspondence analysis (DCA) of the Bray-Curtis metric for each treatment in Study 1. Cattle departed from a feedlot and were transported for a total of 36 h and then either provided 0 h (0H Rest) or 12 h (12H Rest) of rest, followed by an additional 4 h of transportation and unloading at the feedlot. Sampling was conducted before loading (BL), after unloading (AU), 1 day AU, 3 days AU, and 28 days AU.

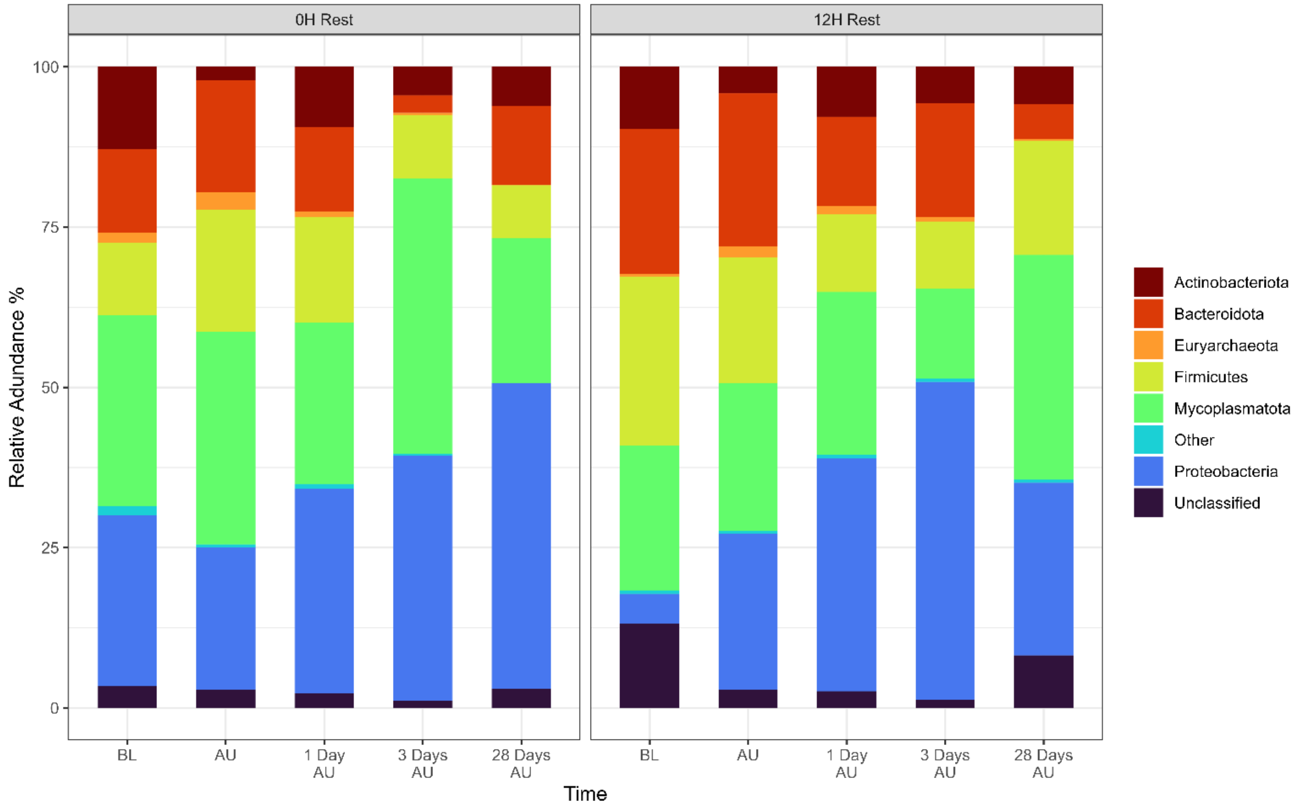

Supplemental Figure 2: Relative abundance of the six most dominant phyla in the nasopharyngeal samples from Study 1. Cattle departed from a feedlot and were transported for a total of 36 h and then either provided 0 h (0H Rest) or 12 h (12H Rest) of rest, followed by an additional 4 h of transportation and unloading at the feedlot. Sampling was conducted before loading (BL), after unloading (AU), 1 day AU, 3 days AU, and 28 days AU. 16S sequences that were assigned to a phylum lacking enough abundance to be visualized were assigned within the 'Other' bar. The 'Unclassified' bar represents the relative abundance of 16S sequences that could not be assigned a phylum level.

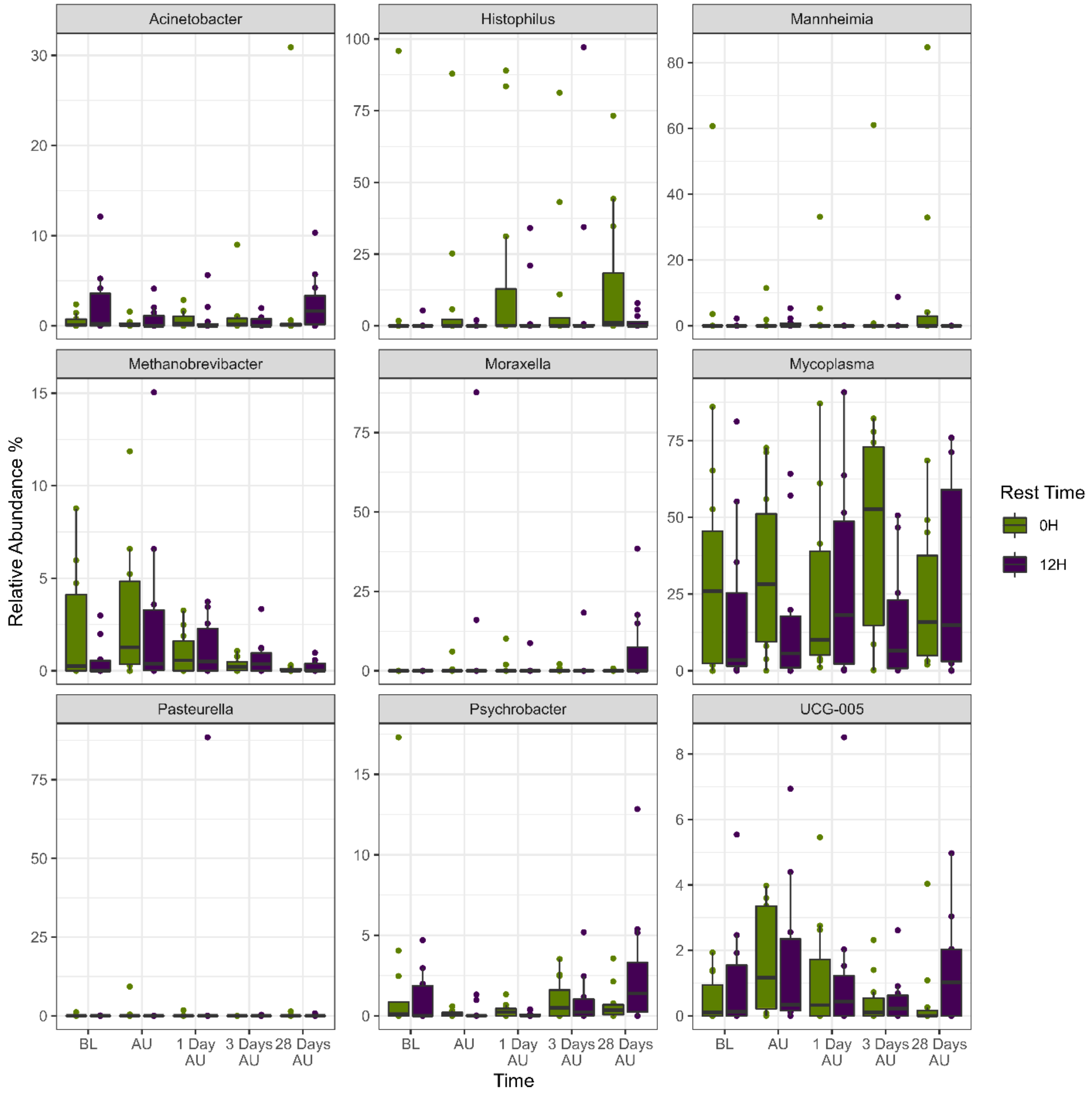

Supplemental Figure 3: Relative abundance of the nine most abundant genera identified in nasopharyngeal swabs from Study 1. Cattle departed from a feedlot and were transported for a total of 36 h and then either provided 0 h (0H Rest) or 12 h (12H Rest) of rest, followed by an additional 4 h of transportation and unloading at the feedlot. Sampling was conducted before loading (BL), after unloading (AU), 1 day AU, 3 days AU, and 28 days AU. The box in the plots indicates the interquartile range (IQR) (middle 50% of the data), the middle line represents the median value, and the whiskers represents 1.5 times the IQR.

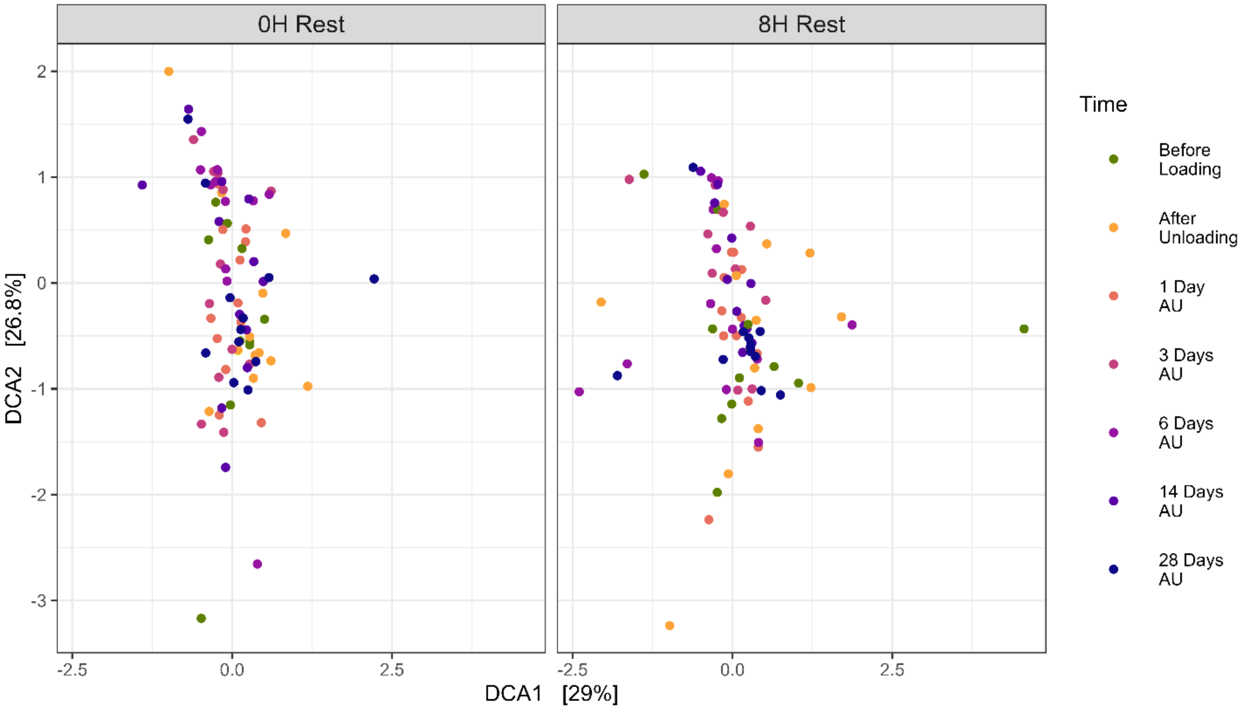

Supplemental Figure 4: Detrended correspondence analysis (DCA) of the Bray-Curtis metric for each treatment in Study 2. Calves from the two treatment groups were loaded onto trailers at the same time, and transported 20 h. Following the assigned rest stop duration (0 h or 8 h), calves were transported an additional 15 h, and then unloaded at the LeRDC feedlot. Nasopharyngeal swabs were collected before loading (BL), after unloading (AU), and then 1, 3, 6, 14, and 28 days AU.

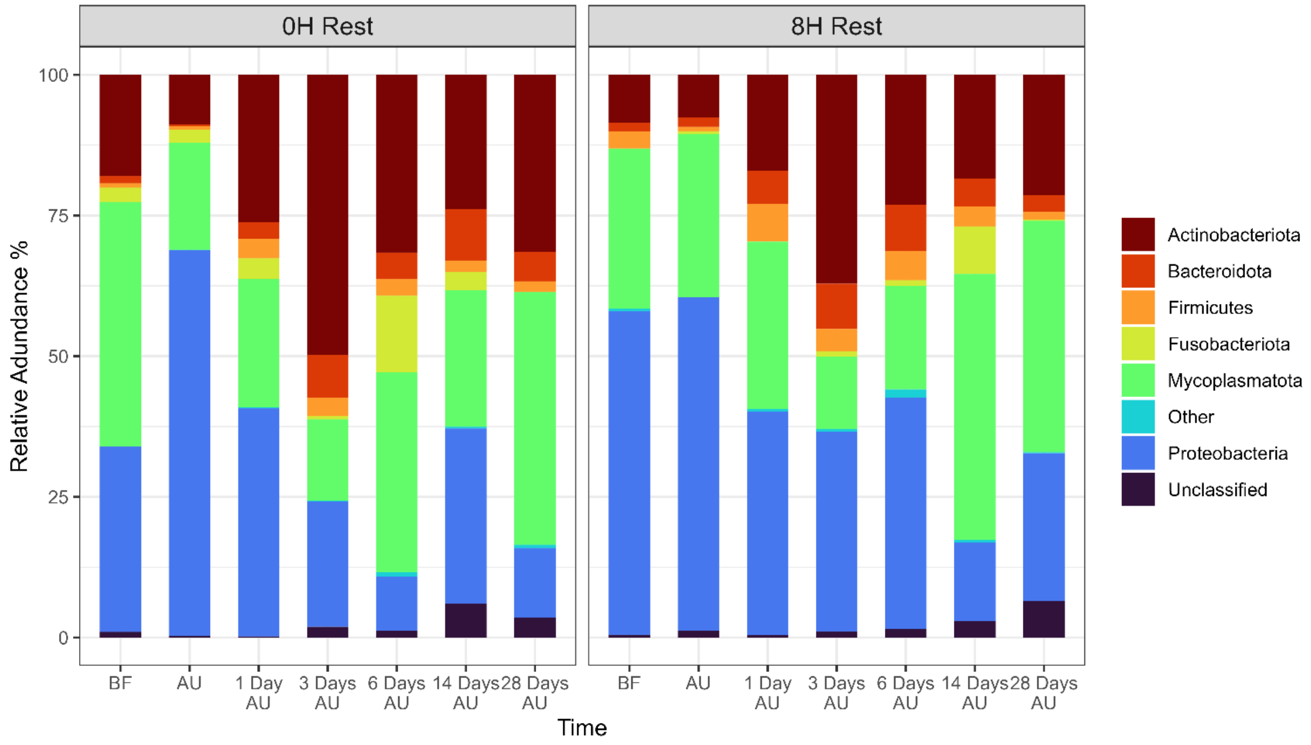

Supplemental Figure 5: Relative abundance of the six most dominant phyla in the nasopharyngeal samples from Study 2. Calves from the two treatment groups were loaded onto trailers at the same time, and transported 20 h. Following the assigned rest stop duration (0 h or 8 h), calves were transported an additional 15 h, and then unloaded at the LeRDC feedlot. Nasopharyngeal swabs were collected before loading (BL), after unloading (AU), and then 1, 3, 6, 14, and 28 days AU. 16S sequences that were assigned to a phylum lacking enough abundance to be visualized were assigned within the 'Other' bar. The 'Unclassified' bar represents the relative abundance of 16S sequences that were unable to be assigned a phylum level.

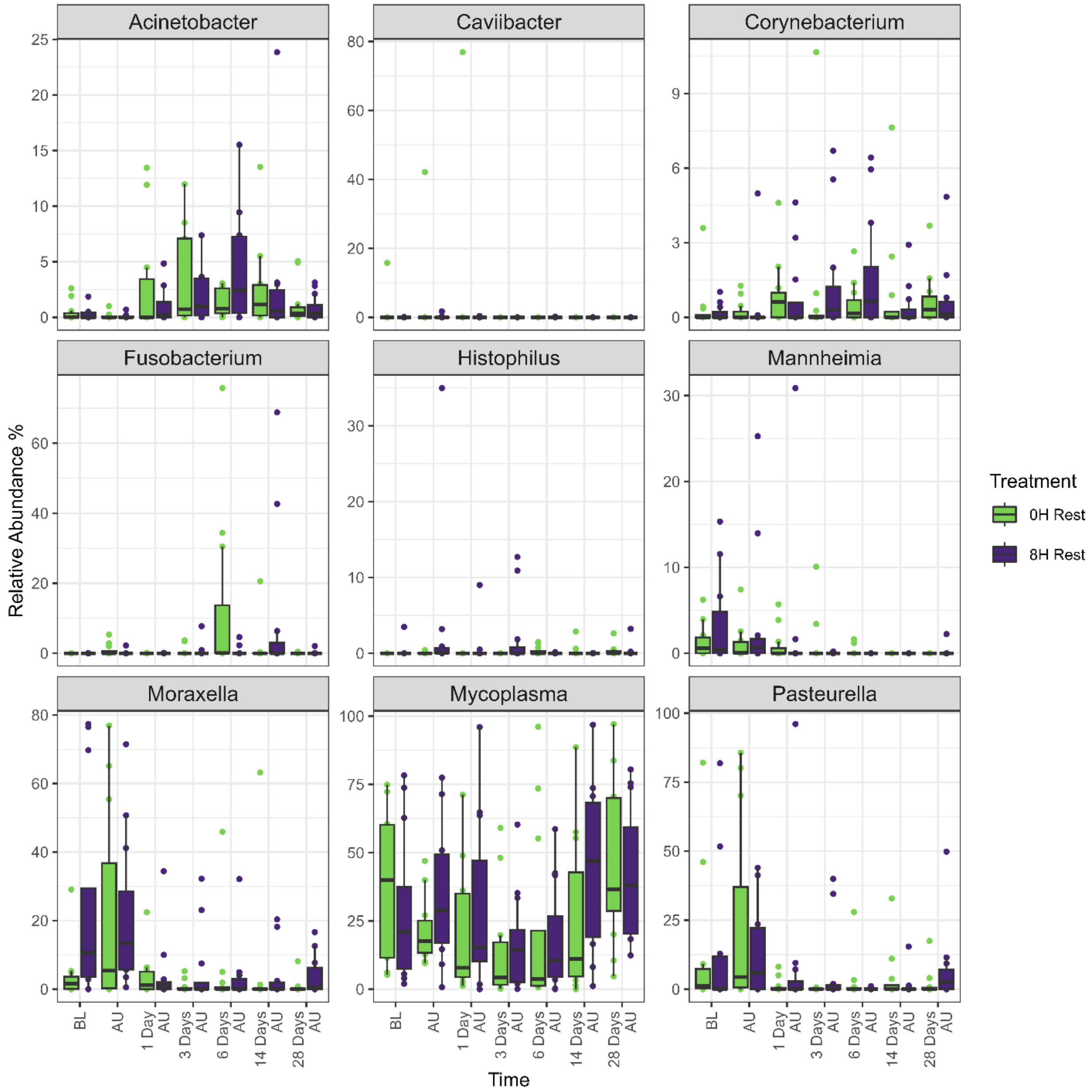

Supplemental Figure 6: Relative abundance of the nine most abundant genera identified in nasopharyngeal swabs from Study 2. Calves from the two treatment groups were loaded onto trailers at the same time, and transported an initial duration of 20 h. Following the assigned rest stop duration (0 h or 8 h), calves were transported an additional 15 h, and then unloaded at the LeRDC feedlot. Samples were collected before loading (BL), after unloading (AU), and then 1, 3, 6, 14, and 28 days AU. The box in the plots indicates the interquartile range (IQR) (middle 50% of the data), the middle line represents the median value, and the whiskers represents 1.5 times the IQR.
